# Restricted Procreation, Introducing a Review of Robinovitch and Others on Specific Human Energy

**Published:** 1910-03

**Authors:** C. H. Hughes

**Affiliations:** St. Louis


### Selected.

### Restricted Procreation, Introducing a Review of Robinovitch and Others on Specific Human Energy.

BY C. H. HUGHES, M. D., ST. LOUIS.

While he of the strenuous life and noble impulse notes with condemnation and regret the tendency to race suicide, but with inadequate qualification, we desire to repeat the record the *Alienist and Neurologist* has sought to make in the direction of commendation rather than condemnation of the prevention of the pre-natal suicide, if one choose to call it so, of the unfit to live, who have yet the procreative power, but can propagate only degeneracy and disease.

The prevention of conception in certain families means the aversion of generations of misery, crime or folly in generations that would follow. One of the inexplicable mysteries connected with the continuation of the human species is the permission, by laws of nature, of the perpetuation of defects by unregulated procreation—the vicious and deformed in mind and body are too often perpetuated after their kind. Morel's table of criminal insane and imbecile degeneracy from original immorality and inebriety and the records of the idiotic Jukes and other families of defectives, fatal to the welfare of society, are standing and potent rebuke and protest against the indifference of mankind to the propagation of that unfit kind whose ways of life from abnormal organic hereditary impulse must lead only to family and society injury. Nature is profligate with the germs of life, being careful only of the type.

A symposium on the treatment of the criminal and other social parasites was held at the joint meeting of the Physicians' Club and the Law Club of Chicago, at the Great Northern Hotel, December

16th of last year, in which elimination of criminal children by sterilization was advocated, and treatment of criminals over 30 years old as habitual malefactors and either sending them to penal institutions for life or putting them away in some other legal manner.

Dr. William T. Belfield was the advocate of the sterilization idea. He took up the biological aspect of the criminal, and after tracing the development of the individual to the point where he became a danger to society, made a plea for race suicide by asexualization.

"Murder, insanity and degeneracy are increasing at an alarming rate," he said. "Our American system of civilization is inadequate to meet the problem of the social parasite. The ignorant hordes of serfs from Southern Europe and Africa have swelled our electorate with those whose ideals are linked with vice and crime. They are filling our city offices with criminals and cementing the alliance between authority and vice. This crude civilization founded on autocracy has come into our democracy during the last twenty-five years.

"Divorce has increased in the same ratio as murder. High finance has developed. The law fixes the punishment of the criminal not according to the danger to the community, but to the individual. The application of the laws of crime is farcical. From a record of the Bureau of Identification I find a colored man fined $85 for trying to assault three little girls.

"Morality is the arrest of the instincts by intellect. A child is a savage. If he continues to improve slowly he has a chance to outgrow his tendencies before he is 30. Before that he should be given all the care possible. But when a man commits a crime after 30 he may be set down, as a rule, to be morally bad with no hope of improvement. For the mature criminal I would advocate permanent segregation either by the penal colony or the cemetery. The indeterminate sentence with the parole is a radical error for the man over 30." Better asexualization before the cemetery.

Dr. J. N. Hurty, Secretary of the Indiana State Board of Health, told of the "Indiana Movement," which is the education of the prisoners in the Indiana Reformatory to allow themselves to be sterilized. More than 300 of them have submitted to this operation so far. The criminals are told who they are and why they are, and after an examination is made of the mental status of the criminal to determine what his chances of improvement are, the operation is performed. Dr. Hurty also told of the operation of the law under which marriage licenses are given.

The *Indiana Medical Journal* thought enough of this important report to select it for reproduction in its pages and we have deemed it worth transcribing.

In a world where so many forms of organic degeneracy and morbid contamination are diffused among the people (syphilitic, alcoholic, gonorrheal, tuberculotic, neurotic, neuropathic, etc.), the question that confronts us with hesitation, apprehension and horror is rather how shall be conserve the yet sound organisms, than diffuse and propagate the unsound and unfit to further continue their defective and degenerate kind upon a world already overburdened with degenerates. What this world needs is more human engines of normal energy, psychical, mental and moral. Every human being represents, as Mm'selle Louise G. Robinovitch has forcefully expressed it, "a certain quantity of potential energies, the greatest part of which is developed and utilized in the direction of genesiac function. The destiny of man is similar to that of every living cell—to live and reproduce itself. It is natural therefore," she says, "to hear a peal of alarm arise when the likelihood of a progressive decrease of reproduction manifests itself in any given nation."

All agree that desirable population should be increased and all must, at least, concede the desirability of eliminating the imbecile, the idiotic, the insanely and otherwise congenitally predisposed defectives and incapables from among a people who would stand strong before the world in good and great accomplishment for their protection and for the welfare of the world. But a high birth rate, as Robinovitch says, "without some definite qualification should be well scrutinized before it is acceptable as a desirable national feature. Limitation of offspring," as she quotes from Dr. Gihon, "in a community where there are 2,000,000 active syphilitics, to say nothing of the other millions unfit for procreation, is often a duty, but fetecide is not its ethical method."

Dr. Alder Blumer, the superintendent of a hospital for the insane, and Dr. M. W. Barr, the superintendent of an institution for imbeciles, are quite in accord, as are alienists and neurologists and all students of morbid cerebropsychology, in favoring legislation for the restraint of reproduction of unfit mental defectives. The experience of Dr. A. W. Wilmarth, of Wisconsin, Superintendent of the State Home for the Feeble-Minded, and Kiernan and all others of similar experience, confirm the fact that defectives are not favorable to race suicide, but prone to plentifully propagate their kind, who have died or are dying out in the highest centers of psychic power. There are circumstances under which the propagation of a human life may be as gravely criminal as the taking of a life already begun and this is only presenting in another and similar phase the view of Blumer, quoted in Robinovitch's valuable contribution, viz., that "the making of a human life is as serious as the taking of one."

Procreation is restricted among some of the economically prudent, according to Dr. Roland Curtin, because of the high price of accouchement and trained nurse services and among the rich and intensely social for selfish considerations of personal convenience, comfort and pleasure. A census, he states, in one of the wealthiest wards in Wilkesbarre, Pa., revealed but a single birth in six months. In another section not even one birth was recorded.

This paper of Robinovitch on which we have so freely drawn and which was read at the Rome Congress of Psychology, in 1905, by the talented editress of the *Journal of Mental Pathology*, has not attracted the general attention its merits, as an original contribution and summary, have deserved.

Procreation is a grave responsibility. Its product is a new unit of energy to the community. If that energy be sexually degenerate, defective and destructive, its product had better be *nil* and absolutely, not partially, impotent, for the good of the world. Pæans sung to procreation and anathemas to prenatal race suicide should be pitched to the proper race conserving key or not sung at all. Prenatal race prevention of the propagation of the unfit is a virtue and not a vice.

The energy of propagation is misdirected and had better be restrained if it brings forth only degeneracy and therefore vital unfitness. And here we introduce and repeat again from the most suggestive, forceful and timely essay already quoted.

"Nations have not yet elevated the energy of genesic function to the dignity of an energy. Other energies known to us, even of the meanest grade, have long since been wisely utilized and their activities based on the principle of the strictest possible economy. This economic utilization has been brought about not through any enforcement of legislative restrictions, but through steadily progressive human intelligence. Economic handling of genesic function will, like the economic function of other energies, come about through a steady and progressive intellectual development of nations." (Robinovitch.)

Many obvious suggestions follow upon this final and novel settlement of fact. One of them is ample provision by the State of accouchement facilities and State borne expense, not for acknowledged paupers, but as a premium upon procreation of non-defective human beings by parents who show the requisite non-defective, organic stamina for the parentage of offspring who will give promise of help and not harm to the commonwealth and that the race in part of their progeny may evolve into the possibilities and responsibilities of higher manhood or womanhood.

A pure food law has passed from a mere possibility to a realized fact. Why not State encouragement of and provision for physically and psychically pure citizens. We restrict immigration because of disease and defect, why not restrict the propagation of defectives among ourselves rather than encourage their harmful multiplication among us?

It were better that the eroto psychopathia engendering seed of the Sodomite die in fecal rectal receptacle or that wombs were fast closed to such, as to the house of Abimelech in Bible story, against another purpose, than that such germs should fructify unto erotopathic degeneracy and dishonor in new beings conceived in utero.

Lot's proposition, it may be here remarked, to give to the perverts of Sodom his virgin daughters, giving them to normal lust in lieu of the sodomy they sought to accomplish, would not be approved by modern psychology, nor would Lot's daughters' method of perpetuating the family name be countenanced, though no epilepsia or other marked psychopathy is recorded as having followed the unconscious and involuntary inebriate incestuous communication, though the Ammonites, however, with their fire god Molech and human sacrifices thereto, appear to have been abnormally pyrophiliac.

The fire and brimstone that rained death from heaven on the persistent Sodomites, was not race suicide, but it accomplished the same salutary purpose against such race contaminations. It was heaven decreed death, according to the record, of the unfit to live and propagate their unfit degenerate kind.

Here is a new vocation for the State, aided by wise medical counsel, for the determination of the fitness or unfitness of two beings by copulation to bring forth a third to become a supporter of self and State and a normal political constituent thereof or an unfit being to burden the State and the family. To avert psychic calamity to a community by preventing the wrong sort of offspring, is better, wiser, more humane and more profitable to family and State than the Spartan method of post-natal extermination or otherwise putting aside as we now do with defectives thrown upon the State for public support.

There is another source of race suicide not quite so germane to our subject as the foregoing matter, wherein nature herself saves the integrity of the race by life prevention and wherein, in contrast with the sun that shines benignly on the just and the unjust alike, the blow of disease falls destructively on the sinless and the sinning, often with greater force on the innocent. I allude to the disease contracted by the man giving the woman a salpingitis which makes her barren and also that other twin venereal scourge that destroys progeny or unfits it for right development into healthful life.

While the novelty of this subject and the method of its treatment may provoke a smile of amusement in some, it is, nevertheless, serious enough in its relation to the mental integrity and perpetuity of good peoples and governments, to challenge the most serious consideration, with efforts at correction by the mentally stable and duly thoughtful physician, philanthropist and patriot.

*The State and Syphilis.*—It is now being urged in many countries by the leaders of medical opinion and social reform that governments should take steps to control the spread of syphilis. This disease is the cause of a vast amount of ill-health and is responsible for many deaths. In the British army it costs the nation about $15,000,000 annually for sickness and about $20,000,000 in pensions and superannuations, or a total of $35,000,000 a year. The disease may be considered from the moral, social and legislative standpoints. The first of these comes more within the range of the church, while the second is one that the medical profession should interest itself in. By legislation the governments of countries might do much by arranging for lectures, the distribution of tracts and the providing of places where syphilis might receive proper treatment. It is certainly a serious blot on our modern civilization that this disease should have free scope to afflict the innocent and guilty alike.

The story of Ross and Edward in the *Medical Fortnightly* for October 10th last, would be approved reading in this connection.

*German Campaign Against Venereal Diseases.*—According to a recent decree of the authorities of Berlin and Charlottenburg, Germany, lectures on hygiene, including sexual hygiene, are to be given regularly in the high schools (gymnasia). The German high schools include part of the college course. The Prussian cabinet has also ordered that instruction in regard to the perils of venereal diseases be imparted to students in the higher institutions of learning. In Darmstadt, the teachers have been ordered to inform the parents of the lectures and to invite them to attend.

*The Peril of the Psycho-Neuropathic Diathesis.*—That other venereal disease to which we have referred in the text as the destroyer of woman's fecundity, happiness and life, quite as formidably claims repressive and eradicative government knowledge and action, even as tuberculosis and other diseases do, but the psychoneuropathic diathesis with its terrible destructive devastation of brains and minds, peopling the insane, idiot, imbecile, inebriate and lunatic institutions and contributing to provide our public schools with backward children, must equally engage popular and official attention if this nation is to be saved.

With enlightened knowledge and resource we now seek to destroy the germs of disease and their carriers, the mosquito, the fly, the flea, the rat, *et id omne genus,* and to purify milk, water and food of disease germs and to destroy other sources and forms of infection and contagion. Why not with like intelligence quarantine against the germs of human degeneracy and decadence?

With scrupulous care the farmer selects his seeds and soil and conducts with equal concern the after care of his growing grain, yet, concerning the seeds and soil of human lives, a strange, unaccountable thoughtlessness too generally prevails. Indifference in this regard too often characterizes human conduct and concern, and conjugal union of creatures who are to engage in the perilous or fortuitous procreation of creatures on whom will devolve the weal or woe of coming generations and the welfare of our States and nations continues as in the days when less was known of the psychopathic peril.

A subscriber asked the editor of a St. Louis city paper to say what is the greatest sin of omission that society is guilty of today.

The editor answered: "It is in the failure of society to realize and provide for the needs of the coming generation."

What would seem to be the supreme desire of humanity—the nurture and protection of its children—is its supreme neglect.

Laws, for instance, that should provide for the restraint and protection of the young are always lowest on the calendar of the legislator.

Why?

Because of the failure or neglect of this generation to appreciate the value of the next.

When attention is urged, the reply is made that the percentage of children who need the help of the State is small. Even were this true—which it is not—it is the bounden duty of the State to look after this percentage.

Millions of children are being well trained for good citizenship in homes and schools.

Other millions—literally millions—are forced to hard labor before thay reach their teens. Other millions are neglected by indifferent, immoral, intemperate or ignorant parents, who roam the streets and engage in every evil.

The State does little for these save to punish them when they are caught.

Take the fact of child labor alone:

It is passing strange that public sympathy should so slowly waken to this monstrous sin.

True, it has few apologists. And in this respect we are better off than during the days of chattel slavery, when its apologists were found in many pulpits. Our sin is spelled in the word neglect.

*Fifty years from now people will look back upon child labor with no less horror than upon the negro slavery of fifty years ago.*

And so it will on the neglect of child heredity and the sin of degenerate propagation.

Byron, the Thanatophobe, said:

> "The skies rain their plagues on men like dew,
> Disease, death, bondage, all the woes we see
> And worse, the woes we see not."

Byron had the death fear of the psychasthenic, that he was at times, from the psychic depression of his erratic excesses and his constitutional hereditary defect of physical organism. The woes we see not included his woe of psycho-neuropathic instability and untimely depression and the unseen woes of those unfortunates who have been dowered and born into the world with a fatal psychopathic heritage, unfit to conquer the adverse ills of life and rightly adapt themselves to their environment. Such had better not have been born. They had better have escaped being born into this life, to them, of unequal contest because of their organically entailed infirmity. They had better have escaped through fortuitous race suicide. —*Alienist and Neurologist.*

---

Peristalsis from left to right, visible through the upper abdomen, is indicative of pyloric obstruction.—*American Journal of Surgery.*

